# Survival in patients on hemodialysis: Effect of gender according to body mass index and creatinine

**DOI:** 10.1371/journal.pone.0196550

**Published:** 2018-05-16

**Authors:** Jeung-Min Park, Jong-Hak Lee, Hye Min Jang, Yeongwoo Park, Yon Su Kim, Shin-Wook Kang, Chul Woo Yang, Nam-Ho Kim, Eugene Kwon, Hyun-Ji Kim, Ji-Eun Lee, Hee-Yeon Jung, Ji-Young Choi, Sun-Hee Park, Chan-Duck Kim, Jang-Hee Cho, Yong-Lim Kim, Clinical Research Center for End Stage Renal Disease (CRC for ESRD) Investigators

**Affiliations:** 1 Department of Internal Medicine, School of Medicine, Kyungpook National University, Daegu, Korea; 2 Clinical Research Center for End Stage Renal Disease in Korea, Daegu, Korea; 3 Department of Internal Medicine, Daegu Fatima Hospital, Daegu, Korea; 4 Department of Statistics, Kyungpook National University, Daegu, Korea; 5 Department of Internal Medicine, Seoul National University College of Medicine, Seoul, Korea; 6 Department of Internal Medicine, Yonsei University College of Medicine, Seoul, Korea; 7 Department of Internal Medicine, College of Medicine, The Catholic University of Korea, Seoul, Korea; 8 Department of Internal Medicine, Chonnam National University Medical School, Gwangju, Korea; Universita degli Studi di Perugia, ITALY

## Abstract

**Background:**

The association of a higher body mass index (BMI) with better survival is a well-known “obesity paradox” in patients on hemodialysis (HD). However, men and women have different body compositions, which could impact the effect of BMI on mortality. We investigated the effect of gender on the obesity-mortality relationship in Korean patients on HD.

**Methods:**

This study included 2,833 maintenance patients on HD from a multicenter prospective cohort study in Korea (NCT00931970). The relationship between categorized BMI and gender-specific mortality was evaluated by an adjusted Cox proportional hazard model with restricted cubic spline analyses and the Competing risk analysis. We also investigated the effect of changes in BMI over 12 months and serum creatinine level on survival in male and female patients on HD.

**Results:**

The mean BMI was 22.6 ± 3.3 kg/m^2^ and the mean follow up duration was 24.2 ± 3.4 months. The patients with the highest quintile of BMI (≥25.1 kg/m^2^) showed lower mortality (subdistributional hazard ratio [SHR] = 0.63, 95% confidence interval [CI] = 0.43–0.93, P = 0.019) compared with those with the reference BMI quintile. When analyzed by gender, male patients with a BMI over 25.1 kg/m^2^ had lower mortality risk (HR = 0.43, 95% CI = 0.25–0.75, P = 0.003); however, no significant difference was found in female patients. Increased BMI after 12 months and high serum creatinine were associated with better survival only in male patients on HD.

**Conclusions:**

BMI could be used as a risk factor for mortality in male patients on HD. However, the mortality of female patients on HD was not related with baseline and follow-up BMI. This suggests that BMI is a good surrogate marker of lean body composition, especially in male patients on HD.

## Introduction

Obesity, defined as a body mass index (BMI) >30 kg/m^2^, is an established risk factor for mortality in the general population [1, 2]. Obesity is an important public health problem in most countries and is associated with metabolic syndrome, which consist of resistance to insulin-stimulated glucose uptake, glucose intolerance, hyperinsulinemia, hypertriglyceridemia, low HDL cholesterol level, and hypertension [3]. In the general population, obesity is associated with increased cardiovascular risk and decreased survival [4].

In contrast, in patients with end-stage renal disease (ESRD), an “obesity paradox” or “reverse epidemiology” (to include lipid and hypertension paradoxes) has been consistently reported and indicates that a higher BMI is paradoxically associated with better survival. The obesity paradox is a universal phenomenon irrespective of race in patients on hemodialysis (HD) [5, 6]. Moreover, maintaining a higher-than-average BMI to preserve “nutritional reserve” may help to reduce the mortality and morbidity rates associated with peritoneal dialysis (PD). However, in Korean patients with PD, a lower BMI was a significant risk factor for death, but increased BMI was not associated with mortality [7].

Regarding gender, men and women have different proportions of skeletal muscle mass and fat mass (body composition) that could affect BMI and serum creatinine level in patients on HD [8]. However, there are few reports on gender and survival in patients on HD according to BMI. Therefore, we hypothesized that gender might affect the relationship between survival and BMI in patients on HD.

In this study, we examined the associations between BMI and mortality in Korean patients on HD. We investigated the association between changes in BMI as a body composition surrogate marker and survival over time. Furthermore, we investigated whether gender affects survival in patients on HD according to body size by subgroup analysis.

## Materials and methods

### Study cohort

We examined data from a nationwide prospective observational cohort study, the End-Stage Renal Disease (ESRD) Registry, from July 1^st^, 2009, to December 31^st^, 2012. During this study period, 2,833 patients on maintenance HD were registered from 31 centers affiliated with the Clinical Research Center for ESRD (CRC for ESRD) (NCT00931970). We excluded 1003 patients with screening failure, those with an uncertain dialysis modality, and those with BMI >36.9 kg/m^2^ or BMI <14.4 kg/m^2^. Finally, we identified 1830 patients on maintenance HD and examined BMI and serum creatinine level from enrollment until 12 months post-enrollment. The CRC registry for ESRD was approved by the medical ethics committees of all participating dialysis centers and informed consent was obtained before inclusion from all patients.

### Ethics statement

The study protocol was approved by Institutional Review Boards of each center before patient enrollment. The names of Institutional Review Boards were as follows. The Catholic University of Korea, Bucheon St. Mary's Hospital; The Catholic University of Korea, Incheon St. Mary's Hospital; The Catholic University of Korea, Seoul St. Mary's Hospital; The Catholic University of Korea, St. Mary's Hospital; The Catholic University of Korea, St. Vincent's Hospital; The Catholic University of Korea, Uijeongbu St. Mary's Hospital; Cheju Halla General Hospital; Chonbuk National University Hospital; Chonnam National University Hospital; Chung-Ang University Medical Center; Chungbuk National University Hospital; Chungnam National University Hospital; Dong A University Medical Center; Ehwa Womens University Medical Center; Fatima Hospital, Daegu; Gachon University Gil Medical Center; Inje University Pusan Paik Hospital; Kyungpook National University Hospital; Kwandong University College of Medicine, Myongji Hospital; National Health Insurance Corporation Ilsan Hospital; National Medical Center; Pusan National University Hospital; Samsung Medical Center, Seoul; Seoul Metropolitan Government, Seoul National University, Boramae Medical Center; Seoul National University Hospital;Seoul National University, Bundang Hospital; Yeungnam University Medical Center; Yonsei University, Severance Hospital; Yonsei University, Gangnam Severance Hospital; Ulsan University Hospital; Wonju Christian Hospital (in alphabetical order). This study was performed in accordance to the 2008 Declaration of Helsinki. Written informed consent was obtained from all patients before inclusion.

### Data collection

Baseline information at the time of enrollment included age, gender, dialysis modality, comorbidities, and laboratory data at the initiation of dialysis. Comorbid conditions included diabetes, congestive heart failure, coronary artery disease, peripheral vascular disease, arrhythmia, cerebrovascular disease, chronic lung disease, peptic ulcer disease, moderate-to-severe chronic liver disease, and malignancy. Laboratory data were available for hemoglobin, blood urea nitrogen (BUN), serum creatinine, albumin, C-reactive protein (CRP), total cholesterol, triglyceride, low density lipoprotein (LDL), and ferritin levels. Dialysis modality was defined as either the modality used 90 days after commencement of dialysis or the initial dialysis modality in patients whose death occurred before 90 days from the commencement of dialysis. The BMI was defined as the body weight divided by the square of the body height, and is universally expressed in units of kg/m^2^. BMI was calculated with dry weight and height at the time of enrollment. The entire range of BMI was divided into the following quintiles: quintile 1, <19.9 kg/m^2^, quintile 2, 19.9–21.6 kg/m^2^, quintile 3, 21.6–23.0 kg/m^2^ (reference range), quintile 4, 23.0–25.1 kg/m^2^, quintile 5, ≥25.1 kg/m^2^. Patient follow-up was censored at the time of death, kidney transplantation, and patient withdrawal. Serum creatinine levels at baseline were divided into the following 5 categories: <6.3 mg/dL, 6.3–8.0 mg/dL, 8.0–9.6 mg/dL, 9.6–11.4 mg/dL, ≥11.4 mg/dL. The relationship between survival rates and changes in BMI over a 12-month follow-up period was assessed by the Competing risk analysis of subdistribution hazard approach with kidney transplantation as competing event to death. The analysis of BMI change was performed only in patients with available data at 1 year after dialysis commencement.

### Clinical and demographic measures

Information on birthdates and first dialysis treatment, gender, all laboratory values, presence of comorbid disease, and dialysis modality was obtained from data collected at the CRC for ESRD. In addition to laboratory values, height and post-HD body weight were also used to calculate BMI.

### Statistical analysis

The baseline characteristics of all patients were compared using the Pearson chi-square test for categorical variables and one-way analysis of variance (ANOVA) with Scheffe posthoc test for continuous variables. The effect of BMI on the time to survival was evaluated first by treating BMI as a quantitative variable, and second by discretizing BMI. For the analysis that regards BMI as a quantitative variable, the relationship between BMI and all-cause mortality was evaluated through a Cox proportional hazard model with restricted cubic spline functions to capture potential nonlinear effects. The adjusted covariates included age, gender, diabetes mellitus as baseline comorbidity, hemoglobin, serum creatinine, albumin, and dialysis vintage. The survival analysis that regards BMI as an ordinal variable was conducted by discretizing the BMI with cut-offs at 19.9, 21.6, 23.0, and 25.1 kg/m^2^, corresponding 20^th^, 40^th^, 60^th^, and 80^th^ percentiles, respectively. Survival curves were estimated by the Kaplan-Meier method and compared by the log-rank test according to BMI category (<25.1 kg/m^2^ and ≥25.1 kg/m^2^). Treating kidney transplantation as competing risk, the analysis based on the subdistribution hazard approach was used to evaluate the effect of BMI category (<25.1 kg/m^2^ and ≥25.1 kg/m^2^) on survival. The Competing risk analysis based on the subdistribution hazard approach was used to calculate the subdistributional hazard ratio (SHR) with 95% confidence interval (CI) for mortality with kidney transplantation as competing event, with BMI range (21.6–23.0 kg/m^2^, third quintile) serving as the reference. Next, we calculated the 1-year changes in BMI and had stratified all patients to 5 groups by BMI change (≥+3 kg/m^2^, +1 to +3 kg/m^2^, -1 to +1 kg/m^2^, -3 to -1 kg/m^2^, and <-3 kg/m^2^). The association between BMI change and mortality with kidney transplantation as competing event was evaluated using the Competing risk analysis with subdistribution hazard approach. Finally, we fit the Competing risk analysis with subdistribution hazard approach to estimate the SHR of mortality with kidney transplantation as competing event for serum creatinine levels at baseline that divided into 5 categories. A *p* value <0.05 was considered statistically significant. SAS for Windows, version 9.3 (SAS Institute Inc., Cary, NC) and R (R Foundation for Statistical Computing, Vienna, Austria; www.r-project.org) were used for statistical analysis.

## Results

### Baseline characteristics across BMI subgroups and association between BMI and mortality

**Fig 1:**
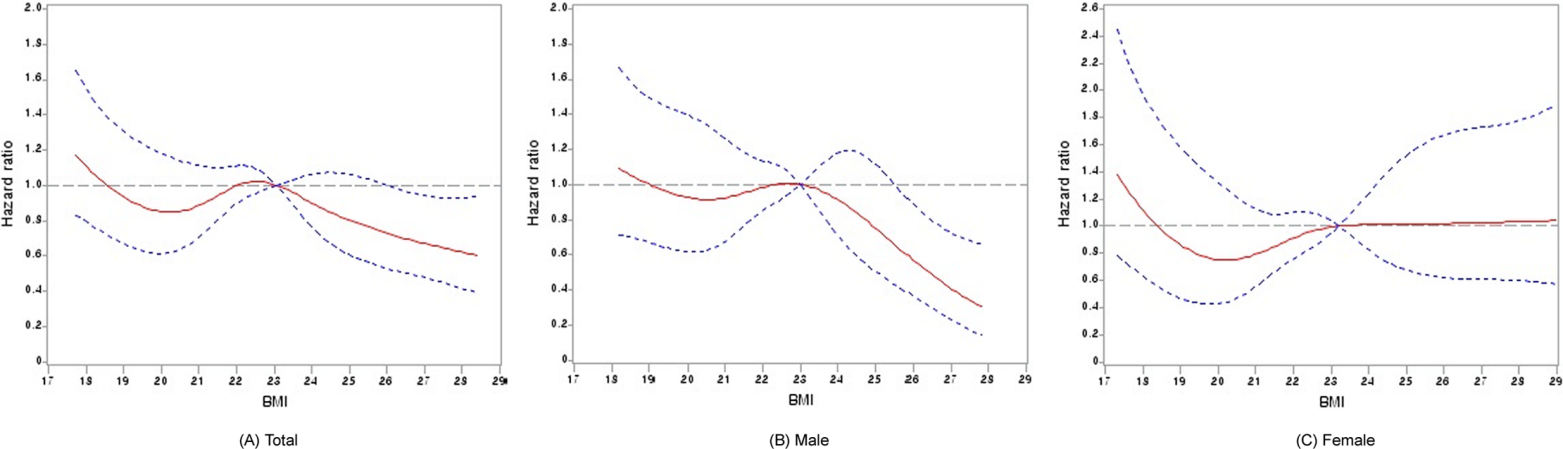
Association between baseline BMI and all-cause mortality hazard ratio in 2,883 Korean patients on maintenance hemodialysis. A Cox regression analysis using restricted cubic splines revealed that higher BMI levels exhibit an association with greater survival in patients. (A) Total (B) Male (C) Female. BMI, body mass index.

The mean BMI was 22.6 ± 3.3 kg/m^2^ and the mean follow-up duration was 24.2 ± 3.4 months. The mean BMI value ranged from 14.4 kg/m^2^ for patients in the lowest BMI quintile to 36.9 kg/m^2^ for patients in the highest BMI quintile. There were significant differences in age, gender, dialysis duration, etiology of ESRD, serum creatinine, triglyceride, and comorbidities including congestive heart failure, cerebrovascular disease, chronic lung disease among the BMI quintiles (S1 Table). Each mean ± standard deviation for BMI quintiles were 18.42 ± 1.18 kg/m^2^ for BMI quintile 1, 20.73 ± 0.48 kg/m^2^ for BMI quintile 2, 22.28 ± 0.42 kg/m^2^ for BMI quintile 3, 22.95 ± 0.61 kg/m^2^ for BMI quintile 4 and 27.49 ± 2.16 kg/m^2^ for BMI quintile 5. A Cox regression analysis using restricted cubic splines was used to evaluate the relationship between BMI and mortality. It revealed that higher BMI levels exhibit an association with greater survival in patients on maintenance HD (Fig 1A).

In multivariate analysis, there was significantly different effect of an interaction among gender and BMI on patient survival in the group of BMI over 25.1 kg/m^2^ (quintile 5) (*p* = 0.030) after adjusted with age, dialysis duration, congestive heart failure, coronary artery disease, peripheral vascular disease, diabetes mellitus, chronic lung disease, moderate to severe liver disease, smoking, albumin, CRP, serum creatinine, total cholesterol, triglyceride, low-density lipoprotein (S2 Table).

### Effect of baseline BMI on survival according to gender

Then, we analyzed the effect of BMI on survival according to gender. Table 1 shows comparison of baseline characteristics between male and female HD patients. The mean BMI was not significantly different (22.6 ± 3.0 kg/m^2^ in men and 22.6 ± 3.6 kg/m^2^ in women). However, there were significant differences in comorbidities including congestive heart failure, coronary artery disease, peripheral vascular disease, chronic lung disease, moderate to severe liver disease, smoking, laboratory data including CRP, serum creatinine, total cholesterol, triglyceride, and LDL between genders (Table 1).

**Table 1:** Baseline characteristics and biochemical data in hemodialysis patients by gender.

	Total (n = 2833)	Male (n = 1647)	Female (n = 1186)	p-value
BMI (kg/m^2)^	22.6±3.3	22.6±3.0	22.6±3.6	0.729
Age (years)	56.1±14.3	56.3±14.3	55.7±14.2	0.307
Etiology to ESRD, n (%)				
Diabetes	1371 (49.3)	824 (51.1)	547 (46.8)	0.172
Hypertension	498 (17.9)	279 (17.3)	219 (18.7)	
Glomerulonephritis	348 (12.5)	194 (12.0)	154 (13.2)	
Others	565 (20.3)	316 (19.6)	249 (21.3)	
Comorbidity, n (%)				
CHF	224 (8.1)	157 (9.7)	67 (5.8)	<0.001
CAD	430 (15.5)	286 (17.8)	144 (12.4)	<0.001
PVD	202 (7.3)	139 (8.6)	63 (5.4)	0.001
Arrhythmia	128 (4.6)	79 (4.9)	49 (4.2)	0.224
CVD	282 (10.1)	171 (10.6)	111 (9.5)	0.196
CLD	224 (8.1)	157 (9.7)	67 (5.8)	<0.001
PUD	184 (6.6)	109 (6.8)	75 (6.4)	0.399
MSLD	106 (3.8)	89 (5.5)	17 (1.5)	<0.001
Malignancy	231 (8.3)	137 (8.5)	94 (8.1)	0.379
Non-smoker (%)	1645 (58.1)	520 (31.6)	1125 (94.9)	<0.001
SBP (mmHg)	141.9±21.4	142.2±20.9	141.5±22.0	0.383
DBP (mmHg)	77.1±13.2	77.0±12.8	77.2±13.8	0.763
Laboratory data				
Hemoglobin (g/dL)	9.9±1.6	9.9±1.7	10.0±1.6	0.253
Albumin (g/dL)	3.7±0.6	3.7±0.6	3.7±0.5	0.776
CRP (mg/dL)	0.4 (0.1–1.6)	0.4 (0.1–1.8)	0.3 (0.0–1.6)	<0.001
s-Cr (mg/dL)	9.0±3.3	9.6±3.5	8.2±2.8	<0.001
TC (mg/dL)	154.3±39.0	147.3±36.8	164.0±39.8	<0.001
Triglyceride (mg/dL)	122.8±80.0	116.2±72.0	131.8±89.1	<0.001
LDL (mg/dL)	85.4±32.6	80.8±30.5	91.8±34.3	<0.001
Ferritin	241.8 (118.0–343.0)	241.8 (120.2–332.3)	241.5 (114.1–362.5)	0.698

Abbreviation: BMI, body mass index; ESRD, end stage renal disease; CHF, congestive heart failure; CAD, coronary artery disease; PVD, peripheral vascular disease; CVD, cerebrovascular disease; CLD, chronic lung disease; PUD, peptic ulcer disease; MSLD, moderate to severe liver disease; SBP, systolic blood pressure; DBP, diastolic blood pressure; s-Cr, serum creatinine; TC, total cholesterol

The patients in the highest quintile for BMI (quintile 5) were associated with greater survival rate than the reference BMI quintile (quintile 3) (SHR 0.63, 95% confidence interval [CI], 0.43–0.93, *p* = 0.019), independently of kidney transplantation by the Competing risk analysis (Table 2). Patients with a BMI over 25.1 kg/m^2^ had a 37% lower mortality risk compared with those with a BMI 21.6 to 23.0 kg/m^2^ (quintile 3, the reference BMI). Additional analysis by gender showed that male patients with a BMI level over 25.1 kg/m^2^ had a 58% lower mortality risk compared with those in the reference BMI quintile (quintile 3) (Fig 1B). However, there was no significant difference in mortality by BMI quintiles in female patients (Fig 1C).

**Table 2:** The association between body mass index quintile and mortality in the Competing risk analysis (n = 2833).

	BMI (kg/m^2)^	Death		KT	
		Adjusted SHR (95% CI)	P	Adjusted SHR (95% CI)	P
Total	<19.9	1.18 (0.84–1.67)	0.340	1.03 (0.55–1.93)	0.940
	19.9 to 21.6	0.86 (0.61–1.22)	0.400	0.95 (0.48–1.88)	0.890
	21.6 to 23	reference			
	23 to 25.1	1.03 (0.74–1.45)	0.850	1.31 (0.69–2.49)	0.400
	≥25.1	0.63 (0.43–0.93)	0.019	0.53 (0.24–1.18)	0.120
Male	<19.9	1.09 (0.71–1.67)	0.700	0.72 (0.33–1.58)	0.410
	19.9 to 21.6	0.87 (0.59–1.29)	0.490	0.66 (0.28–1.54)	0.340
	21.6 to 23	Reference			
	23 to 25.1	0.91 (0.60–1.38)	0.660	1.00 (0.49–2.05)	0.990
	≥25.1	0.42 (0.25–0.72)	0.002	0.61 (0.27–1.38)	0.230
Female	<19.9	1.49 (0.80–2.75)	0.210	2.77 (0.62–12.30)	0.180
	19.9 to 21.6	0.79 (0.39–1.59)	0.510	2.87 (0.63–13.20)	0.180
	21.6 to 23	Reference			
	23 to 25.1	1.26 (0.67–2.37)	0.470	3.26 (0.65–16.50)	0.150
	≥25.1	1.09 (0.58–2.06)	0.790	0.30 (0.02–4.95)	0.400

Abbreviation: BMI, body mass index; KT, kidney transplantation; SHR, subdistribution hazard ratio; CI, confidence interval

Adjusted for age, congestive heart failure, coronary artery disease, peripheral vascular disease, diabetes mellitus, chronic lung disease, moderate to severe liver disease, smoking, albumin, high-sensitivity C-reactive protein, serum creatinine, total cholesterol, triglyceride, low-density lipoprotein, dialysis vintage

**Fig 2:**
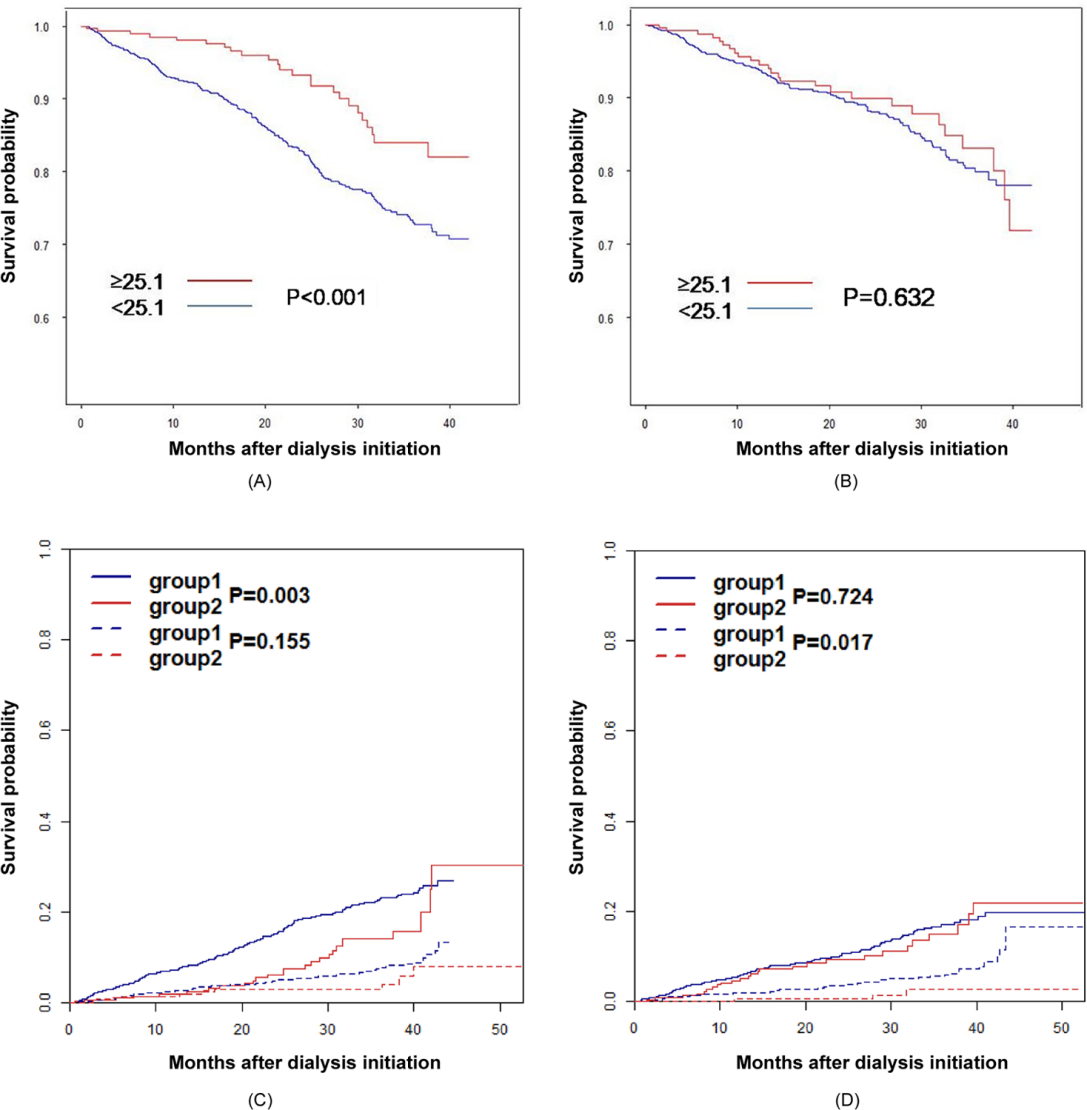
The survival by gender based on a BMI of 25.1 kg/m^2^. On Kaplan-Meier analysis according to BMI 25.1 kg/m^2^ as a reference level, male patients with BMI over 25.1 kg/m^2^ showed greater survival compared to that in patients with a BMI <25.1 kg/m^2^ (p<0.001) (A). Unlike male patients, there was no statistically significant difference in survival among female patients according to BMI (B). On the Competing risk analysis with transplantation as competing event to death, male patients with BMI over 25.1 kg/m^2^ also showed greater survival compared to that in patients with a BMI under 25.1 kg/m^2^ (p = 0.003) (C). In the survival of female patients, there was no statistically significant difference according to BMI on competing risk analysis (D). BMI, body mass index.

On analysis by the Kaplan-Meier method, according to a reference BMI of 25.1 kg/m^2^, male patients with a BMI ≥25.1 kg/m^2^ showed greater survival compared to that in patients with a BMI <25.1 kg/m^2^ (Fig 2). There was no statistically significant difference in the survival among female patients according to BMI.

### Association between BMI change and mortality

Using dry weight measured after 12 months, we examined the association between BMI change and mortality in 1,830 patients on maintenance HD (Table 3). As mentioned previously, we stratified the total patients to 5 groups by BMI change (≥+3, +1 to +3, -1 to +1 kg/m^2^ [reference group], -3 to -1 and <-3 kg/m^2^). The Competing risk analysis according to subdistribution hazard approach with kidney transplantation as competing event showed that a group with a BMI decrease of <-3 was associated with a higher mortality rate (SHR 2.60, 95% confidence interval [CI] 1.16–5.81, *p* = 0.020) compared to that in the reference group. Additionally, we examined the effect of BMI change on mortality by gender. Male patients on HD who lost weight during the 12 months were significantly associated with higher mortality rate (SHR 3.02, 95% confidence interval [CI] 1.26–7.24, *p* = 0.013). No significant difference in mortality according to weight change during the 12 months was found in female patients on HD.

**Table 3:** BMI change and mortality according to BMI quintiles (n = 1830) after 12months.

	Total		Male (n = 1045)		Female (n = 785)	
	Death		Death		Death	
BMI change (kg/m^2^)	SHR (95% CI)	p-value	SHR (95% CI)	p-value	SHR (95% CI)	p-value
≥+3	0.77 (0.10–6.25)	0.810	1.85 (0.20–17.30)	0.590	-	
+1 to +3	1.37 (0.89–2.13)	0.160	1.33 (0.76–2.35)	0.320	1.43 (0.68–3.01)	0.350
-1 to +1	Reference		Reference		Reference	
-3 to -1	1.21 (0.77–1.90)	0.400	1.05 (0.58–1.91)	0.870	1.68 (0.76–3.72)	0.200
<-3	2.60 (1.16–5.81)	0.020	3.02 (1.26–7.24)	0.013	2.49 (0.42–14.70)	0.310

Abbreviation: BMI, body mass index; SHR, subdistributional hazard ratio, confidence interval

Adjusted for age, congestive heart failure, coronary artery disease, peripheral vascular disease, diabetes mellitus, chronic lung disease, moderate to severe liver disease, smoking, albumin, high-sensitivity C-reactive protein, serum creatinine, total cholesterol, triglyceride, low-density lipoprotein, dialysis vintage

Abbreviation: BMI, body mass index; HR, hazard ratio

### Association between creatinine and survival

On stratification of serum creatinine by BMI quintile, quintile 5 (≥11.4 mg/dL) of the highest serum creatinine level at baseline was associated with greater survival compared with quintile 3 (8.0 mg/dL to 9.6 mg/dL) of serum creatinine level as reference (Table 4). In subgroup analysis by gender, male patients on HD in quintile 5 (≥11.4 mg/dL) had greater survival than those in the reference group (SHR 0.38, 95% confidence interval [CI] 0.20–0.74, *p* = 0.004). In contrast to male patients on HD, there was no significant effect on survival by serum creatinine change in female patients on HD.

**Table 4:** Baseline serum creatinine and mortality according to quintile of serum creatinine.

	Total		Male (n = 1045)		Female (n = 785)	
	Death		Death		Death	
s-Cr (mg/dL)	SHR (95% CI)	p-value	SHR (95% CI)	p-value	SHR (95% CI)	p-value
<6.3	1.07 (0.66–1.72)	0.790	1.55 (0.86–2.79)	0.150	0.80 (0.33–1.95)	0.620
6.3 to 8.0	0.70 (0.43–1.15)	0.150	0.50 (0.25–0.97)	0.041	1.28 (0.57–2.86)	0.550
8.0 to 9.6	Reference		Reference		Reference	
9.6 to 11.4	0.72 (0.45–1.14)	0.160	0.66 (0.38–1.14)	0.140	1.13 (0.43–2.96)	0.800
≥11.4	0.47 (0.26–0.84)	0.010	0.38 (0.20–0.74)	0.004	1.12 (0.23–5.37)	0.890

Abbreviation: s-Cr, serum creatinine; SHR, subdistributional hazard ratio; CI, confidence interval

Adjusted for age, congestive heart failure, coronary artery disease, peripheral vascular disease, diabetes mellitus, chronic lung disease, moderate to severe liver disease, smoking, albumin, high-sensitivity C-reactive protein, total cholesterol, triglyceride, low-density lipoprotein, and dialysis vintage

## Discussion

In this multicenter prospective observational cohort study in Korea, we investigated the association between body composition surrogate markers including BMI and serum creatinine and survival in maintenance HD patients and analyzed it by gender in a subgroup analysis. The change of weight as a BMI compound after 12 months was also evaluated for effect on mortality in HD patients. As a result, higher BMI showed better survival in HD patients and statistical significance in male patients, especially. Weight gain after 12 months was associated with better survival in male HD patients but not in female patients on HD.

The human body consists of fat mass, water, and skeletal muscle mass. However, BMI is an imperfect measure of adiposity because it does not differentiate between fat mass and lean body mass (LBM) [9]. LBM can serve as an index of skeletal muscle mass and somatic protein storage, whereas fat mass more directly reflects energy storage. Reduced muscle mass (sarcopenia) is a predictor of mortality in patients on dialysis. As a result, serum creatinine may better reflect muscle mass under steady-state compared to BMI [10]. The lower creatinine level could be a better residual renal function indicator in patients with chronic kidney disease, and could be used as a surrogate of muscle mass, especially in patients with ESRD who have very low residual renal function. By these reasons, many studies about the interaction between body mass and patient survival have used another indicator of body composition along with BMI. We have also used two indices, BMI and serum creatinine, to evaluate the effect of body composition on survival in hemodialysis patients.

BMI and serum creatinine were used as nutritional status indicators and consistently showed a positive correlation with patient survival in many prior reports [1, 11, 12]. They had suggested that higher BMI or higher serum creatinine were significantly correlated with better survival in patients on maintenance HD throughout Europe, Asia, and the United States. Kalantar et al. additionally reported the association of changes in body composition surrogates with survival; a graded relationship with weight loss or gain over time and higher rates of mortality or survival were exhibited, respectively [1]. Park et al. reported a matched cohort study on the association of higher BMI and serum creatinine with greater survival in patients on HD that was consistent across races, especially in a comparison of East Asian and Caucasian and African-American patients [2]. We re-evaluated the obesity paradox prospectively for Korean patients on maintenance HD and assumed that a higher BMI and higher serum creatinine were associated with better survival after adjustment by multivariate analysis. Furthermore, we reassumed that a decrease in BMI over time was associated with poorer survival in patients on maintenance HD.

The obesity paradox is usually explained by a malnutrition-inflammation complex syndrome and protein-energy wasting (PEW) in patients with ESRD [13, 14]. In patients with ESRD, increased PEW is associated with poor prognosis. A nutritional composed scoring system or dietary inflammatory index shows a correlation between PEW and prognosis in patients with ESRD [15, 16]. The pathophysiological mechanisms of PEW have been suggested to be complex including derangements in muscle, adipose tissue, and the gastrointestinal, hematopoietic, and immune systems, complications related to deficiencies of multiple micronutrients, and the maladaptive activation of the inflammatory cascade [17]. Some cytokines such as adiponectin, leptin, circulating actin, gelsolin, and proinflammatory high-density lipoprotein affect PEW in patients with ESRD [17, 18].

Several indices such as age, gender, race, underlying disease, Kt/V, residual renal function (RRF), and nutritional status significantly affect survival in patients with ESRD [19–25]. Among them, clinical indices of nutritional status referring to the ‘obesity paradox’ in patients with ESRD include serum albumin, serum creatinine, normalized protein catabolic rate (nPCR), subjective global assessment (SGA), BMI, mid-arm muscle circumference (MAMC), triceps skin fold (TSF), and LBM [19, 26]. As body mass surrogates, MAMC and TSF represent LBM and fat mass, respectively. Some reports supposed that MAMC as a surrogate of LBM is a potential predictor of quality of life and survival of patients on maintenance HD and may be more important as a surrogate of body fat mass than TSF in predicting survival [10, 27]. Based on this, studies on muscle-gaining interventions in patients on HD, such as intradialytic resistance exercise, were suggested for improving survival in the CKD population [1, 17, 28]. In addition, although some studies on the nutritional status of patients with ESRD have reported evaluation strategies and effective approaches for improving patient survival [25, 29–33], clinical guidelines for improving muscle mass in patients on HD are warranted to promote better clinical outcomes in patients on HD.

Interestingly, out data demonstrated a significantly different effect of BMI on survival by gender on subgroup analysis. According to gender, male patients showed a significant positive association of body composition, BMI, and patient survival. In contrast, female patients had no significant association between body composition, BMI, and survival. The gender difference could be explained by the result of the different effect of sex hormones among genders. Because higher serum testosterone level is associated with increased skeletal muscle mass and reduced fat mass [34], the BMI of male patients may represent skeletal muscle mass more than fat mass proportionally through the effect of serum testosterone. Conversely, female patients, who have relatively lower serum testosterone, may have lower skeletal muscle mass and higher fat mass than male patients [34]. Although patients on HD have decreased sex hormonal level than the general population and the mean age of our study population was 56.1 ± 14.3 years, which mitigates the sex hormonal effect on skeletal muscle mass compared to that in younger patients, serum creatinine as a marker of skeletal muscle mass was higher in male patients on HD than in female patients on HD. This shows a consistent effect of sex hormones on body composition. Therefore, we speculated that the BMI of female patients on HD would have a poorer correlation with skeletal muscle mass than the BMI of male patients; as a result there was no statistically significant effect on female patient survival according to BMI in our study. To prove this hypothesis, we also investigated the association between serum creatinine as a surrogate of skeletal muscle mass and patient survival. The result showed that serum creatinine was significantly associated with patient survival in male patients on HD, but not in female patients. Therefore, the effect of increased body size, especially skeletal muscle mass, on the survival of patients on HD may be different according to gender. Additionally, the method to improve nutritional status of patients on HD would be differently applied according to gender for better survival and quality of life. To prove this, study on the effect of sex hormones on body composition and other contributing factors on survival in patients with HD by gender and gender-specific method improving nutritional status is warranted.

There are some limitations of our study. First, the duration of follow up was short and the results may be limiting for evaluating the effect of BMI and serum creatinine on patient survival. A longer period might show a negative effect of obesity on cardiovascular morbidity and mortality in patients with ESRD such as in the general population. Second, we did not measure the level of sex hormones and evaluate its effectiveness as a body size surrogate; therefore, the effect of sex hormones on body composition in patients with ESRD was not evaluated in our cohort study. Third, on subgroup analysis, we did not evaluate the effect of fat mass, MAMC, and TSF as measures of body composition on survival in patients on HD; a study on this might explain the gender difference. Finally, patients with ESRD on dialysis less than 1 year might have RRF, which could affect patient survival significantly; our study did not adjust for this factor. Nevertheless, to our knowledge, this is the first report on the effect of gender differences on survival by BMI and serum creatinine in patients on maintenance HD.

In conclusion, BMI and serum creatinine could be used as risk factors for mortality in male patients on HD. However, the mortality of female patients on HD was not correlated with baseline and follow-up BMI. This suggests that BMI and serum creatinine are good surrogate markers of lean body composition, especially in male patients on HD.

## Supporting information

S1 Table Baseline characteristics and biochemical data in patients on hemodialysis. (DOCX)

S2 Table Impact of factors on patient survival in multivariate analysis. (DOCX)
