# Supplementary material for: Survival in patients on hemodialysis: Effect of gender according to body mass index and creatinine
Source: PLoS One. 2018 May 16;13(5):e0196550. doi: 10.1371/journal.pone.0196550 (PMC5955527; doi:10.1371/journal.pone.0196550)
Supplement: S1 Table — (DOCX) [file pone.0196550.s001.docx]

**Survival in patients on hemodialysis: effect of gender according to body mass index and creatinine**

Jeung-Min Park^1,8#^, Jong-Hak Lee^2#^, Hye Min Jang^3,8^, Yeongwoo Park^3^, Yon Su Kim^4,8^, Shin-Wook Kang^5,8^, Chul Woo Yang^6,8^, Nam-Ho Kim^7,8^, Eugene Kwon^1,8^, Hyun-Ji Kim^1,8^, Ji-Eun Lee^1,8^, Hee-Yeon Jung^1,8^, Ji-Young Choi^1,8^, Sun-Hee Park^1,8^, Chan-Duck Kim^1,8^, Jang-Hee Cho^1,8*¶^ and Yong-Lim Kim^1,8*¶,^ Clinical Research Center for End Stage Renal Disease (CRC for ESRD) Investigators^^^

^1^ Department of Internal Medicine, School of Medicine, Kyungpook National University, Daegu, Korea,

^2^Department of Internal Medicine, Daegu Fatima Hospital, Daegu, Korea

^3^ Department of Statistics, Kyungpook National University, Daegu, Korea,

^4^ Department of Internal Medicine, Seoul National University College of Medicine, Seoul, Korea,

^5^ Department of Internal Medicine, Yonsei University College of Medicine, Seoul, Korea,

^6^ Department of Internal Medicine, College of Medicine, The Catholic University of Korea, Seoul, Korea,

^7^Department of Internal Medicine, Chonnam National University Medical School, Gwangju, Korea,

^8^ Clinical Research Center for End Stage Renal Disease in Korea, Daegu, Korea

^*^Corresponding Author

E-mail: [ylkim@knu.ac.kr](mailto:ylkim@knu.ac.kr) (YK)

[jh-cho@knu.ac.kr](mailto:jh-cho@knu.ac.kr) (JC)

^#^ Jeung-Min Park and Jong-Hak Lee, these two co-first authors contributed equally to this research.

^¶^ Yong-Lim Kim and Jang-Hee Cho contributed equally to this research.

**S1 Table. Baseline characteristics and biochemical data in patients on hemodialysis**

| BMI quintile  (kg/m^2)^ | Quintile 1  (< 19.9) | Quintile 2  (19.9-21.6) | Quintile 3  (21.6-23.0) | Quintile 4  (23.0-25.1) | Quintile 5  (>25.1) | P-value |
| --- | --- | --- | --- | --- | --- | --- |
| Age (years) | 52.2±16.4 ^a^ | 56.1±14.5 ^b^ | 56.8±13.9 ^b^ | 58.0±12.4 ^b^ | 57.0±13.0 ^b^ | <0.001 |
| Gender (male, %) | 274 (50.4) | 348 (59.6) | 356 (64.3) | 367 (62.6) | 303 (53.4) | <0.001 |
| D-duration (months) | 70.5±67.2 ^c^ | 58.5±55.1 ^b^ | 56.1±57.3 ^b^ | 48.2±44.1 ^a^ | 43.5±42.6 ^a^ | <0.001 |
| Etiology to ESRD, n (%) |  |  |  |  |  |  |
| Diabetes | 199 (36.8) | 278 (48.4) | 248 (47.0) | 306 (52.7) | 340 (24.8) | <0.001 |
| Hypertension | 108 (20.0) | 102 (17.8) | 105 (19.9) | 92 (15.8) | 91 (16.3) | <0.001 |
| Glomerulonephritis | 96 (17.7) | 73 (12.7) | 64 (12.1) | 74 (12.7) | 41 (7.3) | <0.001 |
| Others | 138 (25.5) | 121 (21.1) | 111 (21.0) | 109 (18.8) | 86 (15.4) | <0.001 |
| Comorbidity, n (%) |  |  |  |  |  |  |
| CHF | 75 (14.4) | 71 (12.4) | 58 (11.1) | 52 (9.0) | 55 (9.8) | 0.037 |
| CAD | 72 (13.4) | 95 (16.7) | 86 (16.6) | 80 (13.7) | 97 (17.4) | 0.218 |
| PVD | 36 (6.7) | 40 (7.0) | 42 (8.1) | 43 (7.4) | 41 (7.3) | 0.930 |
| Arrhythmia | 32 (5.9) | 30 (5.2) | 28 (5.4) | 22 (3.8) | 16 (2.9) | 0.087 |
| CVD | 42 (7.8) | 43 (7.4) | 73 (14.0) | 60 (10.4) | 64 (11.4) | 0.001 |
| CLD | 57 (10.6) | 56 (9.7) | 32 (6.2) | 34 (5.9) | 45 (8.0) | 0.012 |
| PUD | 41 (7.6) | 49 (8.5) | 27 (5.2) | 37 (6.4) | 30 (5.3) | 0.121 |
| MSLD | 29 (5.4) | 19 (3.3) | 25 (4.8) | 18 (3.1) | 15 (2.7) | 0.083 |
| Malignancy | 51 (9.5) | 54 (9.4) | 44 (8.5) | 52 (9.0) | 30 (5.3) | 0.070 |
| Non-smoker (%) | 43 (8.1) | 59 (10.4) | 60 (11.6) | 64 (11.2) | 60 (10.8) | 0.360 |
| SBP (mmHg) | 140.4±22.5 | 141.9±21.1 | 140.9±20.9 | 142.8±21.6 | 143.4±20.7 | 0.126 |
| DBP (mmHg) | 77.5±13.9 | 77.1±13.4 | 76.8±13.7 | 76.4±12.7 | 77.6±12.3 | 0.548 |
| Laboratory data |  |  |  |  |  |  |
| Hemoglobin (g/dL) | 10.0±1.5 | 9.9±1.7 | 10.0±1.6 | 9.9±1.8 | 9.8±1.6 | 0.147 |
| Albumin (g/dL) | 3.7±0.6 | 3.7±0.6 | 3.7±0.5 | 3.7±0.5 | 3.6±0.5 | 0.179 |
| CRP (mg/dL) | 2.7±11.0 | 2.3±7.1 | 3.1±12.4 | 3.6±15.0 | 3.9±15.8 | 0.182 |
| s-Cr (mg/dL) | 8.7±2.9 ^a^ | 9.0±3.2 ^a, b^ | 9.2±3.3 ^b^ | 9.2±3.4 ^b^ | 8.8±3.4 ^a^ | 0.026 |
| TC (mg/dL) | 155.2±37.7 | 153.7±38.2 | 154.1±36.7 | 151.9±38.7 | 156.7±43.1 | 0.308 |
| Triglyceride (mg/dL) | 100.9±60.7 ^a^ | 107.5±70.1 ^a^ | 117.4±68.5 ^b^ | 130.6±75.6 ^c^ | 157.4±105 ^d^ | <0.001 |
| LDL (mg/dL) | 85.2±31.9 | 85.2±31.1 | 86.0±33.7 | 84.8±31.9 | 85.9±34.7 | 0.982 |
| Ferritin | 322.7±15.2 | 323.4±15.9 | 292.8±10.9 | 284.1±13.4 | 296.4±12.0 | 0.137 |

Superscripts (a, b, c, d) denote pairs of groups found to be different at the 0.05 level (Scheffe test).

Abbreviation: BMI, body mass index; ESRD, end stage renal disease; D-duration, dialysis duration; CHF, congestive heart failure; CAD, coronary artery disease; PVD, peripheral vascular disease; CVD, cerebrovascular disease; CLD, chronic lung disease; PUD, peptic ulcer disease; MSLD, moderate to severe liver disease; SBP, systolic blood pressure; DBP, diastolic blood pressure; s-Cr, serum creatinine; TC, total cholesterol
