# Supplementary material for: Survival in patients on hemodialysis: Effect of gender according to body mass index and creatinine
Source: PLoS One. 2018 May 16;13(5):e0196550. doi: 10.1371/journal.pone.0196550 (PMC5955527; doi:10.1371/journal.pone.0196550)
Supplement: S2 Table — (DOCX) [file pone.0196550.s002.docx]

**S2 Table. Impact of factors on patient survival in multivariate analysis**

|  | Adjusted Hazard Ratio | Confidence interval | | *P*-value |
| --- | --- | --- | --- | --- |
| Age | 1.049 | 1.037 | 1.037 | <.001 |
| BMI*gender |  |  |  |  |
| 1 |  |  |  | 0.262 |
| 2 |  |  |  | 0.963 |
| 4 |  |  |  | 0.262 |
| 5 |  |  |  | 0.030 |
| CHF | 1.567 | 1.176 | 1.176 | 0.002 |
| CAD | 1.122 | 0.852 | 0.852 | 0.412 |
| PVD | 1.221 | 0.878 | 0.878 | 0.235 |
| DM | 1.146 | 0.891 | 0.891 | 0.289 |
| CLD | 1.244 | 0.891 | 0.891 | 0.200 |
| MSLD | 2.002 | 1.296 | 1.296 | 0.002 |
| Non-smoking | 1.299 | 0.969 | 0.969 | 0.080 |
| Albumin | 0.508 | 0.408 | 0.408 | <.001 |
| CRP | 1.03 | 0.993 | 0.993 | 0.114 |
| s-Cr | 0.938 | 0.895 | 0.895 | 0.007 |
| Total cholesterol | 0.999 | 0.995 | 0.995 | 0.828 |
| TG | 1 | 0.998 | 0.998 | 0.946 |
| LDL | 0.997 | 0.991 | 0.991 | 0.334 |
| Dialysis duration | 1.006 | 1.003 | 1.003 | <.001 |

Abbreviation: BMI, body mass index; D-duration, dialysis duration; CHF, congestive heart failure; CAD, coronary artery disease; PVD, peripheral vascular disease; DM, diabetes mellitus; CLD, chronic lung disease; MSLD, moderate to severe liver disease; CRP, c-reactive protein; s-Cr, serum creatinine; TC, total cholesterol; TG, triglyceride; LDL, low-density lipoprotein
